# Early Estimates of Bivalent mRNA Vaccine Effectiveness in Preventing COVID-19–Associated Hospitalization Among Immunocompetent Adults Aged ≥65 Years — IVY Network, 18 States, September 8–November 30, 2022

**DOI:** 10.15585/mmwr.mm715152e2

**Published:** 2022-12-30

**Authors:** Diya Surie, Jennifer DeCuir, Yuwei Zhu, Manjusha Gaglani, Adit A. Ginde, David J. Douin, H. Keipp Talbot, Jonathan D. Casey, Nicholas M. Mohr, Anne Zepeski, Tresa McNeal, Shekhar Ghamande, Kevin W. Gibbs, D. Clark Files, David N. Hager, Harith Ali, Leyla Taghizadeh, Michelle N. Gong, Amira Mohamed, Nicholas J. Johnson, Jay S. Steingrub, Ithan D. Peltan, Samuel M. Brown, Emily T. Martin, Akram Khan, William S. Bender, Abhijit Duggal, Jennifer G. Wilson, Nida Qadir, Steven Y. Chang, Christopher Mallow, Jennie H. Kwon, Matthew C. Exline, Adam S. Lauring, Nathan I. Shapiro, Cristie Columbus, Natasha Halasa, James D. Chappell, Carlos G. Grijalva, Todd W. Rice, William B. Stubblefield, Adrienne Baughman, Kelsey N. Womack, Jillian P. Rhoads, Kimberly W. Hart, Sydney A. Swan, Nathaniel M. Lewis, Meredith L. McMorrow, Wesley H. Self, IVY Network

**Affiliations:** ^1^National Center for Immunization and Respiratory Diseases, CDC; ^2^Vanderbilt University Medical Center, Nashville, Tennessee; ^3^Baylor Scott & White Health – Baylor Scott & White Medical Center, Temple, Texas; ^4^Texas A&M University College of Medicine, Temple, Texas; ^5^University of Colorado School of Medicine, Aurora, Colorado; ^6^University of Iowa, Iowa City, Iowa; ^7^Wake Forest University Baptist Medical Center, Winston-Salem, North Carolina; ^8^Johns Hopkins Hospital, Baltimore, Maryland; ^9^Hennepin County Medical Center, Minneapolis, Minnesota; ^10^Montefiore Healthcare Center, Albert Einstein College of Medicine, New York, New York; ^11^University of Washington School of Medicine, Seattle, Washington; ^12^Baystate Medical Center, Springfield, Massachusetts; ^13^Intermountain Medical Center and University of Utah, Salt Lake City, Utah; ^14^University of Michigan School of Public Health, Ann Arbor, Michigan; ^15^Oregon Health & Science University Hospital, Portland, Oregon; ^16^Emory University School of Medicine, Atlanta, Georgia; ^17^Cleveland Clinic, Cleveland, Ohio; ^18^Stanford University School of Medicine, Stanford, California; ^19^Ronald Reagan-UCLA Medical Center, Los Angeles, California; ^20^University of Miami, Miami, Florida; ^21^Washington University, St. Louis, Missouri; ^22^The Ohio State University Wexner Medical Center, Columbus, Ohio; ^23^University of Michigan School of Medicine, Ann Arbor, Michigan; ^24^Beth Israel Deaconess Medical Center, Boston, Massachusetts; ^25^Baylor Scott & White Health – Baylor University Medical Center, Dallas, Texas.

Monovalent COVID-19 mRNA vaccines, designed against the ancestral strain of SARS-CoV-2, successfully reduced COVID-19–related morbidity and mortality in the United States and globally (*1*,*2*). However, vaccine effectiveness (VE) against COVID-19–associated hospitalization has declined over time, likely related to a combination of factors, including waning immunity and, with the emergence of the Omicron variant and its sublineages, immune evasion (*3*). To address these factors, on September 1, 2022, the Advisory Committee on Immunization Practices recommended a bivalent COVID-19 mRNA booster (bivalent booster) dose, developed against the spike protein from ancestral SARS-CoV-2 and Omicron BA.4/BA.5 sublineages, for persons who had completed at least a primary COVID-19 vaccination series (with or without monovalent booster doses) ≥2 months earlier (*4*). Data on the effectiveness of a bivalent booster dose against COVID-19 hospitalization in the United States are lacking, including among older adults, who are at highest risk for severe COVID-19–associated illness. During September 8–November 30, 2022, the Investigating Respiratory Viruses in the Acutely Ill (IVY) Network^§^ assessed effectiveness of a bivalent booster dose received after ≥2 doses of monovalent mRNA vaccine against COVID-19–associated hospitalization among immunocompetent adults aged ≥65 years. When compared with unvaccinated persons, VE of a bivalent booster dose received ≥7 days before illness onset (median = 29 days) against COVID-19–associated hospitalization was 84%. Compared with persons who received ≥2 monovalent-only mRNA vaccine doses, relative VE of a bivalent booster dose was 73%. These early findings show that a bivalent booster dose provided strong protection against COVID-19–associated hospitalization in older adults and additional protection among persons with previous monovalent-only mRNA vaccination. All eligible persons, especially adults aged ≥65 years, should receive a bivalent booster dose to maximize protection against COVID-19 hospitalization this winter season. Additional strategies to prevent respiratory illness, such as masking in indoor public spaces, should also be considered, especially in areas where COVID-19 community levels are high (*4*,*5*).

During September 8–November 30, 2022, adults aged ≥65 years admitted for COVID-19–like illness^¶^ to any of 22 hospitals in 18 states participating in the IVY Network were eligible for inclusion in this test-negative design, case-control analysis. Among patients hospitalized with COVID-19–like illness who received testing for SARS-CoV-2 by nucleic acid amplification test or antigen test, those who received a positive test result ≤10 days after illness onset and ≤3 days after hospital admission were classified as case-patients, and those who received a negative test result during the same interval were classified as control patients. Upper respiratory specimens were collected from enrolled patients and retested by reverse transcription–polymerase chain reaction for SARS-CoV-2 and influenza at a central laboratory (Vanderbilt University Medical Center). Patients who were initially enrolled as controls on the basis of negative SARS-CoV-2 test results at their local hospital, but whose test results by central laboratory testing were positive, were reclassified as case-patients in the analysis. Control patients whose influenza test results were positive were excluded from the analysis because of potential correlation between COVID-19 and influenza vaccination behaviors (*6*).

Demographic and clinical data were obtained through electronic medical record (EMR) review and patient (or proxy) interview. COVID-19 mRNA vaccination status was verified from EMR, state-based registries, vaccination cards, or self-report. Three COVID-19 vaccination groups were defined as: 1) unvaccinated (no COVID-19 vaccine doses received); 2) vaccinated with ≥2 monovalent-only mRNA vaccine doses with last dose ≥2 months before illness onset; and 3) vaccinated with ≥2 monovalent-only mRNA vaccine doses plus a bivalent booster dose ≥2 months after receipt of the last monovalent mRNA vaccine dose. Analyses excluded patients with immunocompromising conditions,** those who received a bivalent booster dose <7 days before illness onset or ≤2 months after their last monovalent vaccine dose, those who received a non-mRNA COVID-19 vaccine, and those with other exclusions.^††^

Absolute VE against COVID-19–associated hospitalization was estimated by comparing the odds of bivalent booster dose receipt with no COVID-19 vaccination between case-patients and control patients. Relative VE, which is a measure of the additional protection against COVID-19 hospitalization from a bivalent booster dose compared with residual protection from previous monovalent vaccination, was estimated by comparing the odds of bivalent booster vaccination with receipt of ≥2 monovalent-only mRNA vaccine doses between case-patients and control patients. Relative VE was stratified by number of months (i.e., 2–5, 6–11, or ≥12) between the last monovalent vaccine dose and illness onset. Using multivariable logistic regression models, VE was estimated as (1 − adjusted odds ratio [aOR]) x 100). Models were adjusted for U.S. Department of Health and Human Services region, admission date in 2-week intervals, continuous age, sex, race, and Hispanic or Latino (Hispanic) ethnicity. Estimates with nonoverlapping 95% CIs were considered statistically significant. Analyses were conducted using SAS (version 9.4; SAS Institute). This activity was determined to be public health surveillance by each participating site and CDC, and was conducted consistent with all applicable federal laws and CDC policy.^§§^

During September 8–November 30, 2022, a total of 1,168 immunocompetent adults aged ≥65 years were enrolled in the IVY Network. After exclusion of 370 patients,^¶¶^ 798 (68%) were included in this analysis (381 case-patients and 417 control patients) (Table 1). The median age of included patients was 76 years (IQR = 70–83 years), 118 (15%) were non-Hispanic Black or African American, 78 (10%) were Hispanic, 588 (74%) had at least two underlying conditions, and 66 (8%) had self-reported or documented SARS-CoV-2 infection before the current illness episode during the Omicron period (December 26, 2021–November 30, 2022). Among the 381 case-patients, 81 (21%) were unvaccinated, 280 (73%) had received ≥2 monovalent-only mRNA vaccine doses, and 20 (5%) had received a bivalent booster dose. Among 417 control patients, 62 (15%) were unvaccinated, 296 (71%) had received ≥2 monovalent-only mRNA vaccine doses, and 59 (14%) had received a bivalent booster dose.

**TABLE 1:** Characteristics of immunocompetent adults aged ≥65 years, hospitalized with COVID-like illness,* by COVID-19 case status — IVY Network, 22 hospitals,^†^ 18 U.S. states, September 8, 2022–November 30, 2022

Characteristic	No. (%)		
	Total (N = 798)	COVID-19 case-patients (n = 381)	Test-negative control patients (n = 417)
**Vaccination status**			
Unvaccinated	**143 (18)**	81 (21)	62 (15)
≥2 Monovalent-only mRNA doses	**576 (72)**	280 (73)	296 (71)
Bivalent booster dose^§^	**79 (10)**	20 (5)	59 (14)
**Female sex**	**442 (55)**	210 (55)	232 (56)
**Median age, yrs (IQR)**	**76 (70–83)**	78 (71–85)	75 (69–81)
**Age group, yrs**			
65–74	**345 (43)**	140 (37)	205 (49)
≥75	**453 (57)**	241 (63)	212 (51)
**Race and ethnicity**			
Black or African American, non-Hispanic	**118 (15)**	52 (14)	66 (16)
Hispanic or Latino, any race	**78 (10)**	40 (11)	38 (9)
White, non-Hispanic	**551 (69)**	264 (69)	287 (69)
Other race, non-Hispanic^¶^	**18 (2)**	8 (2)	10 (2)
Other**	**33 (4)**	17 (4)	16 (4)
**HHS region^†^**			
1	**155 (19)**	91 (24)	64 (15)
2	**50 (6)**	29 (8)	21 (5)
3	**9 (1)**	4 (1)	5 (1)
4	**94 (12)**	40 (11)	54 (13)
5	**125 (16)**	66 (17)	59 (14)
6	**99 (12)**	42 (11)	57 (14)
7	**68 (9)**	27 (7)	41 (10)
8	**145 (18)**	58 (15)	87 (21)
9	**29 (4)**	14 (4)	15 (4)
10	**24 (3)**	10 (3)	14 (3)
**No. of underlying medical conditions**			
0	**38 (5)**	15 (4)	23 (6)
1	**172 (22)**	91 (24)	81 (19)
2	**243 (30)**	115 (30)	128 (31)
≥3	**345 (43)**	160 (42)	185 (44)
**Previous Omicron infection^††^**	**66 (8)**	24 (6)	42 (10)

**Abbreviation:** HHS = U.S. Department of Health and Human Services.

* COVID-19–like illness was defined as including any one of the following: fever, cough, shortness of breath, new or worsening findings on chest imaging consistent with pneumonia, or hypoxemia defined as oxygen saturation (SpO2) <92% on room air or supplemental oxygen to maintain SpO2 ≥92%. For patients on chronic oxygen therapy, hypoxemia was defined as SpO2 below baseline or an escalation of supplemental oxygen to maintain a baseline SpO2.

^†^ Hospitals by HHS region included *Region 1:* Baystate Medical Center (Springfield, Massachusetts) and Beth Israel Deaconess Medical Center (Boston, Massachusetts); *Region 2:* Montefiore Medical Center (New York, New York); *Region 3:* Johns Hopkins Hospital (Baltimore, Maryland); *Region 4:* Emory University Medical Center (Atlanta, Georgia), University of Miami Medical Center (Miami, Florida), Vanderbilt University Medical Center (Nashville, Tennessee), and Wake Forest University Baptist Medical Center (Winston-Salem, North Carolina); *Region 5:* Cleveland Clinic (Cleveland, Ohio), Hennepin County Medical Center (Minneapolis, Minnesota), The Ohio State University Wexner Medical Center (Columbus, Ohio), and University of Michigan Hospital (Ann Arbor, Michigan); *Region 6:* Baylor Scott & White Health – Baylor Scott & White Medical Center (Temple, Texas) and Baylor Scott & White Health – Baylor University Medical Center (Dallas, Texas); *Region 7:* Barnes-Jewish Hospital (St. Louis, Missouri) and University of Iowa Hospitals (Iowa City, Iowa); *Region 8:* Intermountain Medical Center (Murray, Utah) and UCHealth University of Colorado Hospital (Aurora, Colorado); *Region 9:* Stanford University Medical Center (Stanford, California) and UCLA Medical Center (Los Angeles, California); and *Region 10:* Oregon Health & Science University Hospital (Portland, Oregon) and University of Washington (Seattle, Washington).

^§^ Bivalent COVID-19 mRNA booster dose recipients received ≥2 monovalent COVID-19 mRNA doses ≥2 months before their bivalent booster dose.

^¶^ Other race, non-Hispanic includes Asian, Native American or Alaska Native, and Native Hawaiian or other Pacific Islander; these groups were combined because of small counts.

** Self-reported race and ethnicity as other, or patients for whom information on race and ethnicity was unavailable.

^††^ Previous Omicron infection was defined by date of self-reported or documented previous SARS-CoV-2 infection that occurred during December 26, 2021–November 30, 2022.

The median interval between receipt of a bivalent booster dose and illness onset was 29 days (IQR = 15–45 days) (Table 2). When compared with unvaccinated patients, VE of a bivalent booster dose in preventing COVID-19–associated hospitalization was 84%. When compared with patients who had received ≥2 monovalent-only mRNA vaccine doses ≥2 months before illness onset, relative VE of a bivalent booster dose was 73%. When compared with patients whose last monovalent dose was 6–11 months and ≥12 months before illness onset, relative VE of a bivalent booster dose was 78% and 83%, respectively. Small sample size precluded estimation of the relative VE of a bivalent booster dose compared with receipt of ≥2 monovalent-only mRNA vaccine doses with last dose 2–5 months before illness onset.***

**TABLE 2:** Effectiveness of a bivalent COVID-19 mRNA booster dose against COVID-19–associated hospitalization among immunocompetent adults aged ≥65 years — IVY Network, 22 hospitals,* 18 states, September 8, 2022–November 30, 2022

Characteristic	Received BV vaccine dose, by case status, n/N (%)		Median interval^†^ from last vaccine dose to illness onset (IQR), days	Adjusted VE, % (95% CI)^§^
	Case-patients	Control patients		
**Absolute VE (BV booster dose versus no vaccine)**				
Unvaccinated (Ref)	—	—	NA	—
BV booster dose^¶^ ≥7 days before illness onset	20/101 (20)	59/121 (49)	29 (15–45)	84 (64–93)
**Relative VE (BV booster dose versus MV-only, by interval since last dose)**				
≥2 MV-only mRNA doses, last dose ≥2 mos before illness onset (Ref)	—	—	305 (168–377)	—
BV booster dose ≥7 days before illness onset	20/300 (7)	59/355 (17)	29 (15–45)	73 (52–85)
≥2 MV-only mRNA doses, last dose 2–5 mos before illness onset (Ref)			137 (111–155)	—
BV booster dose ≥7 days before illness onset	20/82 (24)	59/155 (38)	29 (15–45)	—**
≥2 MV-only mRNA doses, last dose 6–11 mos before illness onset (Ref)	—	—	304 (258–333)	—
BV booster dose ≥7 days before illness onset	20/155 (13)	59/176 (34)	29 (15–45)	78 (57–89)
≥2 MV-only mRNA doses, last dose ≥12 mos before illness onset (Ref)	—	—	528 (386–575)	—
BV booster dose ≥7 days before illness onset	20/103 (19)	59/142 (42)	29 (15–45)	83 (63–92)

**Abbreviations:** BV = bivalent; MV = monovalent; NA = not applicable; Ref = referent group; VE = vaccine effectiveness.

* The IVY Network includes the following hospitals: Baystate Medical Center (Springfield, Massachusetts), Beth Israel Deaconess Medical Center (Boston, Massachusetts), Montefiore Medical Center (New York, New York), Vanderbilt University Medical Center (Nashville, Tennessee), University of Miami Medical Center (Miami, Florida), Emory University Medical Center (Atlanta, Georgia), Johns Hopkins Hospital (Baltimore, Maryland), Wake Forest University Baptist Medical Center (Winston-Salem, North Carolina), Baylor Scott & White Health – Baylor Scott & White Medical Center (Temple, Texas), University of Iowa Hospitals (Iowa City, Iowa), University of Michigan Hospital (Ann Arbor, Michigan), Hennepin County Medical Center (Minneapolis, Minnesota), Barnes-Jewish Hospital (St. Louis, Missouri), Cleveland Clinic (Cleveland, Ohio), The Ohio State University Wexner Medical Center (Columbus, Ohio), Stanford University Medical Center (Stanford, California), UCLA Medical Center (Los Angeles, California), UCHealth University of Colorado Hospital (Aurora, Colorado), Oregon Health & Sciences University Hospital (Portland, Oregon), Intermountain Medical Center (Murray, Utah), University of Washington (Seattle, Washington), and Baylor Scott & White Health – Baylor University Medical Center (Dallas, Texas).

^†^ For patients who received a BV booster dose, median time since last dose refers to the number of days between receipt of the BV booster dose and illness onset. For patients who received ≥2 MV doses without a BV booster dose, median time since last dose refers to the number of days between receipt of the last MV dose and illness onset.

^§^ VE was estimated by comparing the odds of being BV-vaccinated among case-patients to the odds of being BV-vaccinated among control patients, calculated as VE = 100 × (1 – odds ratio). Logistic regression models were adjusted for date of hospital admission (biweekly intervals), U.S. Department of Health and Human Services (10 regions), continuous age, sex, and race and ethnicity (non-Hispanic Black or African American, Hispanic of any race, non-Hispanic White, non-Hispanic other race, or other or unknown).

^¶^ BV COVID-19 mRNA booster dose recipients received ≥2 MV COVID-19 mRNA doses ≥2 months before their BV booster dose.

** VE estimate was not reported because of insufficient sample size. 95% CI width >50 percentage points.

## Discussion

Among immunocompetent adults aged ≥65 years hospitalized within the IVY Network in 18 states, a bivalent booster dose received after ≥2 monovalent mRNA doses provided strong protection against COVID-19–associated hospitalization during a period of Omicron BA.5 or BQ.1/BQ.1.1 predominance (*7*). Substantial additional protection from a bivalent booster dose was observed when compared with remote monovalent-only mRNA vaccination, which suggests important incremental benefit for persons eligible to receive a bivalent vaccine booster. These early findings from a cohort of adults aged ≥65 years, 74% of whom had multiple underlying conditions, are among the first to document real-world evidence that receipt of a bivalent booster dose after completion of at least a primary COVID-19 mRNA vaccination series is protective against COVID-19 hospitalization. Continued monitoring will be important to understand ongoing protection in the context of expanding Omicron sublineages and new emerging variants, as well as whether waning of bivalent vaccine-induced immunity over time is observed, similar to that seen after monovalent COVID-19 mRNA vaccine booster doses.

Recent findings from the United Kingdom and the United States have also demonstrated protection of a bivalent mRNA booster dose against COVID-19 hospitalization (*8*,*9*). The bivalent mRNA booster vaccine used in the United Kingdom contains spike protein mRNA from ancestral SARS-CoV-2 plus Omicron BA.1, in contrast to the bivalent booster vaccines used in the United States, which contain mRNA from ancestral SARS-CoV-2 and Omicron BA.4/BA.5. In the United Kingdom, among adults aged ≥50 years or those in clinical risk groups, a BA.1 bivalent booster dose was found to have a relative VE of 57% (95% CI = 48%–65%) compared with ≥2 COVID-19 vaccine doses received ≥6 months earlier (*8*). Similarly, a report among adults aged ≥18 years from the VISION Network in the United States using BA.4/BA.5 bivalent booster doses showed a relative VE of 42% (95% CI = 19%–58%) against COVID-19–associated hospitalization compared with ≥2 monovalent COVID-19 vaccine doses received 8–10 months earlier (*9*). Overall, these results were similar to the relative VE findings in the current study, suggesting that bivalent booster doses provide important benefits.

The findings in this report are subject to at least five limitations. First, the sample size was not sufficient to estimate VE by the number of COVID-19 monovalent vaccine doses received before the bivalent booster dose or compared with patients whose most recent monovalent vaccine dose was received 2–5 months before illness onset. Second, because use of monovalent COVID-19 mRNA vaccines as a booster dose is no longer authorized in the United States,^†††^ this analysis could not compare the effectiveness of a bivalent booster dose with a monovalent booster dose administered during the same period. Third, the analysis period includes both BA.5- and BQ.1/BQ.1.1–predominant periods; therefore, variant-specific VE could not be evaluated. Fourth, previous SARS-CoV-2 infection during the Omicron period was rarely reported or documented among patients in this analysis, which prevented evaluation of the impact of previous infection on VE. Finally, selection bias and residual confounding bias cannot be excluded, including from risk behaviors or preventive treatments.

These early findings from a multistate network show that among adults aged ≥65 years, many of whom have multiple comorbid conditions and who are at highest risk of severe COVID-19, recent bivalent booster vaccination offers substantial added protection against COVID-19 hospitalization. Although prevention of COVID-19 hospitalizations is a core goal of the U.S. vaccination program, bivalent booster dose coverage in the United States remains low among adults aged ≥18 years (16%) and adults aged ≥65 years (36%) (*10*). Increasing bivalent booster coverage among eligible U.S. adults has the potential to prevent COVID-19 hospitalizations as COVID-19 incidence and transmission increase. All eligible persons, especially adults aged ≥65 years, should receive a bivalent booster dose to maximize protection against COVID-19 hospitalization this winter season. Additional strategies to prevent respiratory illness, such as masking in indoor public spaces, should also be considered, especially in areas where COVID-19 community levels are high (*4*,*5*).

**Summary**

**What is already known about this topic?**

Immunity from monovalent COVID-19 mRNA vaccination wanes over time. A bivalent COVID-19 mRNA booster dose is recommended for all eligible persons; however, little is known about its effectiveness against COVID-19 hospitalization.

**What is added by this report?**

Among immunocompetent adults aged ≥65 years hospitalized in the multistate IVY Network, a bivalent booster dose provided 73% additional protection against COVID-19 hospitalization compared with past monovalent mRNA vaccination only.

**What are the implications for public health practice?**

To maximize protection against severe COVID-19 this winter season, all eligible persons, especially adults aged ≥65 years, should receive a bivalent booster dose and consider additional prevention strategies, including masking in indoor public spaces.

## Footnotes

* These authors contributed equally to this report.

† These senior authors contributed equally to this report.

§ The IVY Network includes the following hospitals: Baystate Medical Center (Springfield, Massachusetts), Beth Israel Deaconess Medical Center (Boston, Massachusetts), Montefiore Medical Center (New York, New York), Vanderbilt University Medical Center (Nashville, Tennessee), University of Miami Medical Center (Miami, Florida), Emory University Medical Center (Atlanta, Georgia), Johns Hopkins Hospital (Baltimore, Maryland), Wake Forest University Baptist Medical Center (Winston-Salem, North Carolina), Baylor Scott & White Health – Baylor Scott & White Medical Center (Temple, Texas), University of Iowa Hospitals (Iowa City, Iowa), University of Michigan Hospital (Ann Arbor, Michigan), Hennepin County Medical Center (Minneapolis, Minnesota), Barnes-Jewish Hospital (St. Louis, Missouri), Cleveland Clinic (Cleveland, Ohio), The Ohio State University Wexner Medical Center (Columbus, Ohio), Stanford University Medical Center (Stanford, California), UCLA Medical Center (Los Angeles, California), UCHealth University of Colorado Hospital (Aurora, Colorado), Oregon Health & Sciences University Hospital (Portland, Oregon), Intermountain Medical Center (Murray, Utah), University of Washington (Seattle, Washington), and Baylor Scott & White Health – Baylor University Medical Center (Dallas, Texas).

¶ COVID-19–like illness was defined as including any one of the following: fever, cough, shortness of breath, new or worsening findings on chest imaging consistent with pneumonia, or hypoxemia defined as oxygen saturation (SpO2) <92% on room air or supplemental oxygen to maintain SpO2 ≥92%. For patients on chronic oxygen therapy, hypoxemia was defined as SpO2 below baseline or an escalation of supplemental oxygen to maintain a baseline SpO2.

** Immunocompromising conditions were defined as active solid tumor or hematologic cancer (i.e., newly diagnosed cancer or cancer treatment within the previous 6 months); solid organ transplant; bone marrow or stem cell transplant; HIV infection; congenital immunodeficiency syndrome; use of an immunosuppressive medication within the previous 30 days; splenectomy; or another condition that causes moderate or severe immunosuppression.

†† Exclusions: 1) immunocompromising conditions; 2) illness onset after hospital admission; 3) enrollment >7 days after hospital admission; 4) SARS-CoV-2-positive test result >3 days after hospital admission; 5) co-infection with influenza or respiratory syncytial virus (RSV); 6) positive influenza test result in control patients; 7) receipt of non-mRNA vaccine; 8) partial vaccination (receipt of only 1 mRNA vaccine dose); 9) receipt of last monovalent vaccine dose <2 months before illness onset; 10) receipt of bivalent vaccine dose <2 months after last monovalent dose; or 11) withdrawal from study.

§§ 45 C.F.R. part 46.102(l)(2), 21 C.F.R. part 56; 42 U.S.C. ^§^241(d); 5 U.S.C. ^§^552a; 44 U.S.C. ^§^3501 et seq.

¶¶ A total of 370 patients were excluded from the analysis for the following reasons (not mutually exclusive): patient did not meet COVID-19–like illness definition (two); illness onset occurred after hospital admission (12); patient enrolled >7 days after hospital admission (21); inability to obtain an upper respiratory sample for central laboratory testing among controls (19); SARS-CoV-2 test >3 days after hospital admission (three); SARS-CoV-2 testing indeterminate (seven); case-patient received a positive influenza test result (three); control patient received a positive influenza test result (100); influenza testing indeterminate or not done (42); case-patient received a positive RSV test result (five); inability to verify vaccination status (71); received non-mRNA vaccine (66); partial vaccination (30); received last monovalent dose <2 months before illness onset (10); received bivalent COVID-19 mRNA booster dose <2 months after last monovalent dose (three); received COVID-19 vaccines outside of CDC guidelines (13); other sex (two); or withdrew (two).

*** VE estimates with 95% CIs >50 percentage points were not reported because of lack of precision.

††† https://www.fda.gov/news-events/press-announcements/coronavirus-covid-19-update-fda-authorizes-moderna-pfizer-biontech-bivalent-covid-19-vaccines-use (Accessed December 9, 2022).
